# Characterization of Inducible Transcription and Translation-Competent HIV-1 Using the RNAscope ISH Technology at a Single-Cell Resolution

**DOI:** 10.3389/fmicb.2018.02358

**Published:** 2018-10-02

**Authors:** Wang Zhang, Sara Svensson Akusjärvi, Anders Sönnerborg, Ujjwal Neogi

**Affiliations:** ^1^Division of Clinical Microbiology, Department of Laboratory Medicine, Karolinska Institutet, Solna, Sweden; ^2^Science for Life Laboratory, Division of Proteomics and Nanobiotechnology, KTH Royal Institute of Technology, Solna, Sweden; ^3^Department of Medicine Huddinge, Unit of Infectious Diseases, Karolinska Institutet, Karolinska University Hospital, Solna, Sweden

**Keywords:** RNAscope, RNAflow, HIV-1 latency, latency-reversing agents, single-cell characterization

## Abstract

Identifying the source and dynamics of persistent HIV-1 at single-cell resolution during cART is crucial for the design of strategies to eliminate the latent HIV-1 reservoir. An assay to measure latent HIV-1 that can distinguish inducible from defective proviruses with high precision is essential to evaluate the efficacy of HIV-1 cure efforts but is presently lacking. The primary aim of this study was therefore to identify transcription and translation competent latently infected cells through detection of biomolecules that are dependent on transcriptional activation of the provirus. We investigated the applicability of two commercially available assays; PrimeFlow^TM^ RNA Assay (RNAflow) and RNAscope^®^ ISH (RNAscope) for evaluation of the efficacy of latency reversal agents (LRAs) to reactivate the HIV-1 latent reservoir. The J-Lat cell model (clones 6.3, 9.3, and 10.6) and four LRAs was used to evaluate the sensitivity, specificity, and lower detection limit of the RNAflow and RNAscope assays for the detection and description of the translation-competent HIV-1 reservoir. We also checked for HIV-1 subtype specificity of the RNAscope assay using patient-derived subtype A1, B, C, and CRF01_AE recombinant plasmids following transfection in 293T cells and the applicability of the method in patient-derived peripheral blood mononuclear cells (PBMCs). The lower detection limit of RNAflow was 575 HIV-1 infected cells/million and 45 cells/million for RNAscope. The RNAscope probes, designed for HIV-1B, also detected other subtypes (A1, B, C, and CRF01_AE). RNAscope was applicable for the detection of HIV-1 in patient-derived PBMCs following LRA activation. In conclusion, our study showed that RNAscope can be used to quantify the number of directly observed individual cells expressing HIV-1 mRNA following LRA activation. Therefore, it can be a useful tool for characterization of translation-competent HIV-1 in latently infected cell at single-cell resolution in the fields of HIV-1 pathogenesis and viral persistence.

## Introduction

From a clinical perspective, three read-outs in HIV-cure research can be considered: latency reversal, reservoir reduction and viral remission after cessation of combinational antiretroviral therapy (cART) (Churchill et al., 2016). Understanding the size of the reservoir and how best to target it, is a stepping-stone for HIV-cure research. Identifying the source and dynamics of persistent HIV-1 at single-cell resolution during cART is crucial for understanding the barriers for a functional HIV cure. A primary challenge in HIV-cure research is development of robust assays that can quantify the true reservoir of integrated, replication-competent provirus within the host cellular DNA and strategies that can eliminate or control this persistent viral reservoir.

An assay to measure HIV-1 latency that can distinguish inducible from defective proviruses with high precision is essential for evaluation of efficacy of eradication efforts but is presently lacking. Quantitative HIV-1 DNA PCR assays measure all proviruses, of which the vast majority are defective (Ho et al., 2013), thereby overestimating the size of the latent replication-competent reservoir. The quantitative viral outgrowth assay (Q-VOA), termed as the “golden-standard”, measures induced proviruses (Karlsson et al., 2001; Laird et al., 2013); however, it underestimates the size of the reservoir as it is unable to detect intact non-induced provirus (Eriksson et al., 2013). The technique TILDA (Tat/rev Induced Limiting Dilution Assay) measures the frequency of cells with inducible, multiply-spliced HIV-1 RNA that is absent in latently infected cells but can be induced upon viral reactivation (Procopio et al., 2015). However, large discrepancies are found between these assays that results in a significant hurdle for clinical trials that aim to measure the efficacy of HIV-1 eradication strategies (Bruner et al., 2015).

Recent studies have shown that the RNA-flow fluorescent *in situ* hybridization (FISH) technique can be useful to measure the translation or transcription-competent reservoir with high sensitivity and specificity (Baxter et al., 2017; Grau-Exposito et al., 2017). The method can also provide relevant information when studying HIV pathogenesis, persistence, and reactivation. Further, a more recent study using the branched DNA *in situ* hybridization technology combined with multiplex immunofluorescent cell-based detection of DNA, RNA, and Protein (MICDDRP) targeting both HIV-1 RNA, integrated provirus and viral proteins indicated that the method can be applied at single-cell resolution (Puray-Chavez et al., 2017).

In this study, we investigated the applicability of two commercially available assays, PrimeFlow^TM^ RNA Assay (herein RNAflow) and RNAscope^®^ ISH technique (herein RNAscope) for evaluation of the efficacy of latency reversal agents (LRAs) to induce the HIV-1 latent reservoir. For this purpose, the J-Lat cell model and low input of patients’ peripheral blood mononuclear cells (PBMCs) were used, respectively. We also checked the HIV-1 subtype specificity of the assays. Our study indicated that due to the loss of a large number of cells in RNAflow, the RNAscope technique perform better than RNAflow. In addition, RNAscope had a lower detection limit while scanning 1 × 10^6^ cells, independent on the patient-derived HIV-1 subtype (HIV-1A1, HIV-1B, HIV-1C, 01_AE).

## Materials and Methods

### Optimization of Latency-Reversing Agents (LRAs) to Achieve Maximal Reactivation of HIV-1 Latency

To optimize the reversal capacity of different LRAs, three J-Lat cell models of HIV latency (J-Lat Full Length Clones 6.3, 9.2, 10.6, NIH AIDS Reagent Program, United States) were used (Jordan et al., 2003). These cells are modified to contain integrated GFP replacing *nef* within the proviral genome. The parental cell line, Jurkat was used as negative control (Weiss et al., 1984). Cells were cultured in RPMI medium (10% fetal bovine serum (FBS, Gibco, Life Technologies, United States), 0.2% Penicillin-Streptomycin (Penstep, Gibco, Life Technologies, United States) and 0.2% Plasmocin^TM^ prophylactic (InvivoGen, United States) and stimulated using individual and combinations of LRAs. The LRAs were tested in each cell model at final concentration of the Protein kinase C (PKC) agonists 12-deoxyphorbol-13-acetate (Prostratin, 6 μM, Sigma-Aldrich, MO, United States); human TNFα (hTNFα; 10 ng/mL, Thermo Fisher, United States); Calcium ionophore (Ionomycin, 1.25 μM, Sigma-Aldrich, MO, United States) and Suberoylanilide hydroxamic acid (SAHA, 6.25 μM, Sigma-Aldrich, United States), known to inhibit Histone deacetylase (HDACi). The exposure time of LRAs was standardized to 48 h. Reactivation of latent provirus and cell viability were monitored using GFP expression and viability dye (LIVE/DEAD^TM^ Fixable Aqua Dead Cell Stain Kit, Thermo Fisher, United States) using flow cytometry (BD FACSVerse^TM^, United States).

### Surface Modified Slides and Coverslips

To prevent de-attachment of cells from coverslips during RNAscope, we used different surface modified slides, e.g., H-12-Collagen, H12-PDL, GG-12-Laminin, GG-12-Fibronectin, GG-12-Gelatin (Neuvitro Corporation, Vancouver, United States) and poly-L-lysin coverslips (Corning, United States). The poly-L-lysin coverslips provided better attachment and fluorescence signal during imaging and were therefore used for RNAscope.

### HIV-1 mRNA Detection Using RNAflow

Cells were exposed for 48 hrs using LRAs followed by PrimeFlow^TM^ (PrimeFlow RNA assay, Affymetrix/eBioscience, United States) detection of HIV-1 mRNA by flow cytometry, according to the manufacturer’s protocol. Forty probe pairs were used, 20 targeting *gag* and 20 targeting *pol*. Samples were collected in tubes followed by fixation and permeabilization. Probes were diluted and hybridized to the target mRNA for 2 h at 40°C. Samples were washed to remove excess probes, and signal amplification was achieved by sequential incubations with the pre-amplification and amplification mix (1.5 h at 40°C each). Amplified mRNA was labeled with Alexa Fluor 647-tagged probes (1 h at 40°C). Samples were acquired on a FACSVerse (BD Bioscience, United States) and analysis performed using FlowJo (Treestar, V10, United States). Un-activated/healthy control samples were used as gating control. Normalized positive cell per million (NPM) were calculated by the formula: NPM = (RNAflow^+^ cells/DAPI^+^ cells) × 10^6^. All the experiments were performed at least two technical replicates with three biological replicates.

### HIV-1 mRNA Detection Using RNAscope

RNAscope^®^ (RNAscope^®^ Fluorescent Multiplexed reagent kit, Advanced Cell Diagnostics, United States) was used as per the manufacturer’s protocol and adjusted for dual detection of mRNA and protein while retaining maximal amount of cells/coverslip. The probe set, V-HIV1-clade B-C3 (Advanced Cell Diagnostics, United States), consisted of 20 dual probes targeting different segments within the *gag-pol* region. During all preparation stages, coverslips were handled gently using forceps with no high velocities upon the coverslips as it greatly affected the attachment of cells. After activation, cells were washed two times 5 min using PBS at 1500 rpm prior attachment of 500,000 cells in 100 μL on Bio-coat pre-coated poly-L-lysin coverslips (Corning, United States) 30 min at 37°C. Un-attached cells were removed using PBS prior fixation using Formalin Solution, neutral buffered 10%, (NBF, Sigma-Aldrich, United States) for 20 min at RT and washed two times 5 min PBS. Pre-treatment for storage was performed by de-hydration using EtOH, 50, 70, and 100% for 2 min at room temperature subsequently for storage in 100% EtOH at -20°C for up to 1 month. Storage beyond 1 month decreases the signal. For re-hydration, the opposite concentration decrease of EtOH was performed with a final 10 min equilibrium in PBS. Cells were permeabilized using 0.1% PBS Triton-x for 10 min at RT prior washing and storing coverslips in PBS, 1 min. Coverslips were attached on Superfrost glass slides (Thermo Fisher, United States) using nail polish, and a hydrophobic barrier created using Immedge^TM^ Hydrophobic Barrier Pen (Vector Laboratory, United States). Probe hybridization was achieved by incubation of 35 μL mRNA target probes for 2 h at 40°C using a HyBez oven. The signal was amplified by subsequent incubation of Amp-1, Amp-2, Amp-3 and Amp4 (Alexa Fluor 647-tagged probe), one drop each for 30, 15, 30, and 15 min respectively at 40°C using a HyBez oven. Each incubation step was followed by two times 2 min wash using RNAscope washing buffer in slide holders with agitation (50 rpm). Glass slides were always applied into slide holder containing washing buffer, cells upward using forceps. Nucleic acids were stained using manufacturers supplied 4′,6′-diamino-2-phenylindole (DAPI, Advanced Cell Diagnostics, United States) for 30 s and washed two times PBS, if not followed by subsequent protein staining, as described below, prior nucleic acid stain. The coverslips were mounted on Superfrost^TM^ Plus Adhesion Slides (Thermo Fisher, MA) using Prolong Gold Antifade Mountant (Thermo Fisher, United States). Unless otherwise stated, big field images of the whole coverslip were taken with a Nikon inverted confocal microscope equipped with ×20/0.75 objective and high-speed camera (Andor Zyla 4.2+, Belfast, Northern Ireland) utilizing the tiling function. The excitation/emission bandpass wavelengths used to detect DAPI, GFP/FITC and Alexa 647 were set to 405/425–480, 488/503–555 and 647/655–705 nm, respectively. Using the latest NIS Element Software with the function for object identification and automatic counting, HIV^mRNA+/GFP+^ cells were identified. Normalized positive cell per million (NPM) were calculated by the formula: NPM = (RNA^+^ cells /GFP^+^ cells/ DAPI^+^ cells)×10^6^. Super-resolution images were captured using a Nikon Single point scanning confocal microscope with ×40/0.95 and × 60/1.4 oil objective.

### Immunofluorescence Staining for HIV-1 p24

Staining for HIV p24 protein was performed after RNAscope and before staining for nucleic acids. Cells were blocked using 1% bovine serum albumin (BSA) in 0.1% PBST (Tween20) or 5% milk in 0.1% PBST at RT for 30 min followed by antibody (Ab) labeling using FITC tagged anti-p24 antibody (10 μg/ml, Biolegend, United States) or Ms anti-HIV-1 p24 Ab (1 μg/mL, Abcam, United Kingdom) at RT for 1 h/over-night at 4°C respectively. FITC stained samples were washed twice in PBST at RT for 10 min with agitation. For secondary Ab labeling samples were washed twice in PBST at RT for 5 min on agitation prior labeling with anti-Ms IgG H&L Alexa 488 (Abcam, United Kingdom).

### Specificity of RNAflow and RNAscope

Reactivated J-Lat 10.6 cells were mixed with parental Jurkat cells in 10-fold dilutions from 10 to 0.001% with the addition of 20%. Samples were then analyzed using RNAflow or RNAscope. The percentage of J-Lat 10.6 cells, determined by dilution scale, were used for prediction of NPM for each sample in the dilution series. Detected values were compared to the predicted values to determine the linearity and Limit of Detection (LOD) of the assay. LOD was calculated with the corresponding linear equation and y = mean_blank_ + 3SD_blank_. All the experiments were performed at least three technical replicates with three biological replicates.

Specificity of RNAflow and RNAscope were tested using three controls; negative control (J.Lat 10.6^Activated-/Probe+^), technical negative control (J.Lat 10.6^Activated+/Probe-^) and positive control (J.Lat 10.6^Activated+/Probe+^).

### RNAscope to Detect Diverse HIV-1 Subtypes RNA

To evaluate the specificity of RNAscope to HIV-1 group M subtypes, specific clones (A1, B, C, 01_AE and 02_AG) were isolated from stored plasma samples of randomly chosen adult patients at Karolinska University Hospital, Stockholm, Sweden, and used for transfection of HEK 293T cells. The pNL4-3 plasmid was used as a positive control (NIH AIDS reagent program, United States). The subtypes were confirmed to be pure (HIV-1A1, HIV-1B, and HIV-1C) or circulating recombinant form (01_AE and 02_AG) by near full-length genome sequencing (Aralaguppe et al., 2016).

### Cloning of Patient-Derived HIV-1 Gag-Pol

Viral RNA was extracted from 140 μL plasma using QIAmp Viral RNA Mini Kit (Qiagen, Germany). For a generation of the complementary strand, superscript III RT enzyme (Invitrogen, United States) was used with the gene-specific primer 6231R, as described by our group (Grossmann et al., 2015). The high-fidelity KAPA HiFiHotStart Ready Mix (KAPA Biosystem, United States) was used for two rounds of PCR. The first-round primers were 0682F and 6231R. Second-round primers 0702F-*BssHII* (*GCGCGC*CTAGAAGGAGAGAGAGATGGGTGCGAG) and 5798R-*SalI (GTCGAC*CTCTCATTGCCACTGTCTTCTGCTC) contained the restrictions sites *BssHII* and *SalI* (New England Biolabs, United States) respectively that were subsequently used for cloning in the pNL4-3Δ*gag-pol* plasmid and validated by sequencing as described recently (Neogi et al., 2018).

### Transfection With Clones From HIV-1 Subtypes

Patient-derived clones were transfected in HEK 293T cells in a 1:3 dilution of DNA: Fugene (Promega, United States) using 1 μg DNA for 24 h. All transfection reagents were mixed in Opti-MEM^TM^ (Gibco, Life Technologies, Carlsbad, United States) to a proportion of 1:10 of total media volume of RPMI. After 24 h post-transfection, media was removed and cells washed twice in PBS prior fixation using 10% NBF for 20 min at RT. Subsequently, RNAscope was performed and IF using primary and secondary antibody staining. Images were acquired using single point scanning confocal microscopy.

### PBMCs Isolation and Detection

As a proof-of-concept, EDTA-blood samples were obtained from treatment naïve HIV-1 infected patients with viremia (*n* = 2) as well as HIV-1 negative individuals (*n* = 2). Patient 1 (PT#01) was infected with CRF01_AE and had a plasma viremia of 1970000 copies/mL while patient 2 (PT#02) was infected with HIV-1C with a plasma viremia of 69700 copies/mL. PBMCs were isolated by using Ficoll-Paque Plus reagent (GE Healthcare Life Sciences, Piscataway, NJ) according to the manufacturer’s protocol. Cells were frozen in 10% dimethyl sulfoxide (DMSO)–90% FBS after isolation. Upon preparation, donor PBMCs were allowed to recover 24 hrs in RPMI (10% FBS, 0.2% Penstrep and 20 units IL-2 (1 μg/mL, PeproTech). After recovery, LRA activation was conducted prior RNAscope procedure, including IF staining using FITC tagged Ab, as described above, to determine the NPM value. The experiments were performed two technical replicates with two biological replicates.

### Ethical Considerations

The study was approved by regional ethics committees of Stockholm (2013/1944–31/4). All participants gave informed consent, and patients identity was anonymized and delinked before analysis.

## Results

**FIGURE 1:**
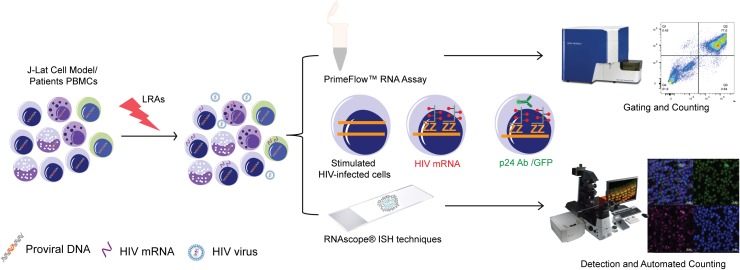
Study designing and experimental plan: Schematic representation of experimental plan for RNAflow and RNAscope.

There are currently no reliable markers for a precise detection and quantification of latently infected cells *ex vivo* nor *in vitro*. Studies aiming at quantifying the proportion of transcription and translation competent latently infected cells through biomolecule detection are dependent on transcriptional activation of the provirus. We explored the capacity of mRNA detection *in situ* to detect HIV-1 transcripts by flow cytometry (RNAflow) and microscopy (RNAscope) to evaluate the sensitivity and specificity of each detection system following latency reversal using LRAs (**Figure 1**).

### LRAs Optimization

**FIGURE 2:**
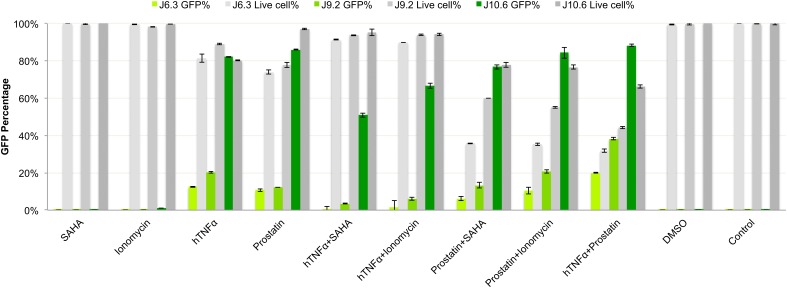
Optimization of latency reversing reagents (LRAs). Three J-Lat cell models 6.3, 9.2, and 10.6 were used to screen for optimal LRA combinations. The doses used were: hTNFα: 10 ng/ml, Prostratin: 6 μM, Ionomycin: 1.25 μM, SAHA: 6.25 μM as a single agent or in combinations. Percentage of GFP positive cells and viability were measured. J-Lat 10.6 showed maximum activation and was further used for RNA detection using the RNAflow and RNAscope. The LRA combinations hTNF-α and prostatin showed maximum activation in all three-cell model and further used as cLRAs.

In our study, the optimal combination of LRAs for reactivation of latent HIV-1 was Prostatin (6 μM) and hTNFα (10 ng/mL), as measured by GFP expression, and cell viability (**Figure 2**). In J-Lat6.3 and J-Lat9.2, hTNFα + Prostatin (20 and 38.2% respectively) hade a significant increase in activation (*p* < 0.05) compared to hTNFα/SAHA (0.6 and 3.5% respectively), hTNFα/Ionomycin (1.5 and 6.2% respectively), Prostatin/SAHA (6.18 and 13.3% respectively) and Prostatin/Ionomycin (10.3 and 21% respectively) while in J-Lat10.6 hTNFα + Prostatin (88%) hade a more significant increase in activation than hTNFα/SAHA (51%) and hTNFα/Ionomycin (66.7%). This combination LRA (cLRA) was therefore used for all experiments.

### Optimization of RNAscope Workflow to Improve Cell Recovery Rate

Extensive washing steps tend to affect the overall output of both RNAflow and RNAscope. For RNAflow we observed ∼70–82% of cell loss while performing all assays in the low-binding Eppendorf tube, while RNAscope procedure contributed to a ∼70–78% loss of cells on coverslips following the centrifugation in the CytoSpin *centrifuge* (Thermo Fisher, United States). To improve cell coverage, we aimed at optimizing the coating strategy of coverslips to gain sufficient electrostatic interactions between cell membrane (negatively charged ions) and coverslips (positively charged ions) using Poly-L-lysine, without changing the 3D morphology. Poly-L-Lysine pre-treatment positively affected the cell coverage shown by an increase in the attachment of activated J-Lat 10.6 cells, as measured by GFP (**Supplementary Figure 1A**). The optimization induced an increase in cell retention from 22.4 to 85.9%, from the input number of cells after the complete RNAscope procedure (**Supplementary Figure 1B**). Poly-L-Lysine is a strong coating agent that mediates an increase in positively charged sites available for binding on the coated surface thereby retaining cells during multiple washing steps.

### Specificity and Limit of Detection (LOD) of RNAflow in Latently Infected Cells

**FIGURE 3:**
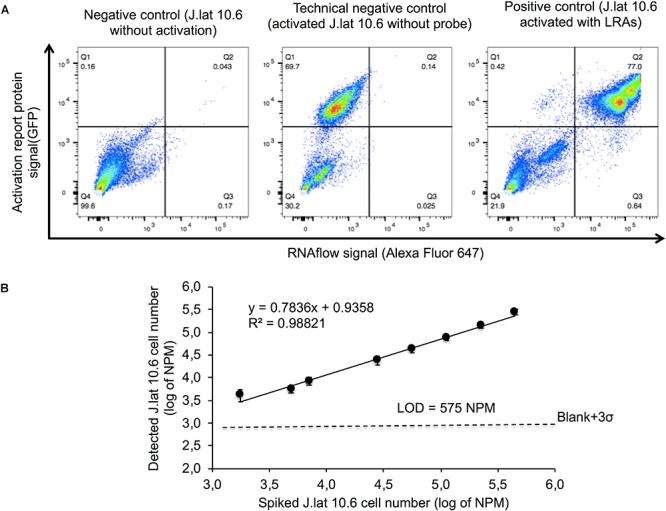
Gating strategy and determination of the lower detection limit of RNAflow. Reactivated J-Lat 10.6 cells were spiked into parent Jurkat cells in 10-fold dilutions from 10% to 0.001% with the addition of 20% population. **(A)** The gating strategy for RNA positive cells (lower right panel), GFP and RNA positive cells (upper right panel) and GFP positive cells (upper left panel). Specificity of RNAflow were tested using three controls; negative control (J.Lat 10.6^Activated-/Probe+^), technical negative control (J.Lat 10.6^Activated+/Probe-^) and positive control (J.Lat 10.6^Activated+/Probe+^). **(B)** Detected values were compared to the predicted values to determine the linearity and Limit of Detection (LOD) of the assay which was calculated using the corresponding linear equation (y = mean_blank_ + 3SD_blank_).

The quantified proportion of activated HIV^mRNA+/GFP+^ cells using PrimeFlow^TM^ was 77% of total cells compared to 0.043 and 0.14% detection in the negative and technical controls, respectively. HIV^mRNA-/GFP-^ cells were detected at a rate of 21.9, 99.6, and 30.2% in J-Lat 10.6^Activation+/Probe+^, J-Lat 10.6^Activation-/Probe+^ and J-Lat 10.6^Activation+/Probe-^, respectively (**Figure 3A**). The LOD of the PrimeFlow assay was determined to be 575 normalized cell numbers/million (NPM) (**Figure 3B**). One of the major disadvantages of using RNAflow is the high cell loss during the preparational stages. We intended to analyze 1 × 10^6^ cells. Following RNAflow, we were able to acquire 1 × 10^5^ to 2 × 10^5^ cells with an input of 1 × 10^6^ cells due to cell loss. The protocol required more than 15-fold centrifugation steps with a reduction in cell numbers for each wash. Thus, as latently infected cells can be <100 cells in one million the starting material would have to be extremely large to facilitate any reliable data. Due to the limitations in accessibility of patient material and low sensitivity, PrimeFlow^TM^ was not an advantageous option for this application.

### Specificity and Limit of Detection (LOD) of RNAscope in Latently Infected Cells

**FIGURE 4:**
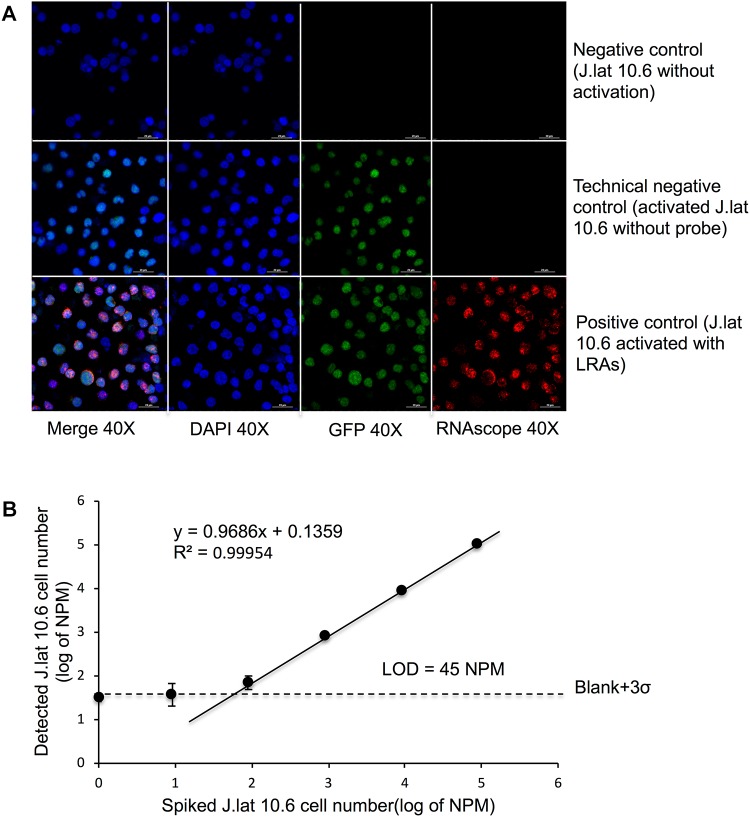
Automated counting strategy and the lower detection limit of RNAscope **(A)** Detection of rare cells by microscopy following RNAscope HIV^mRNA+/GFP+^, HIV^mRNA+^ and HIV^GFP+^ cells were detected after LRA stimulation using confocal microscopy with a series (as mentioned in **Figure 3**) of activated J-Lat 10.6 cells in the parental Jurkat cell line **(B)** LOD was measured by automatic cell counting and analysis for dual recognition of HIV^mRNA+/GFP+^ using the same formula mentioned in **Figure 3**. Automated counting detected all mRNA signal related to cells that have an active transcription of GFP, and correlates it to the proportion of all detected nuclei (DAPI).

Detection of rare cells by microscopy have been made available by RNAscope due to its high sensitivity and specificity as probes and reagents are optimized to yield a low background. Here HIV^mRNA+/GFP+^ could easily be identified after stimulation with LRAs using confocal microscopy (**Figure 4A**). Co-localization of the protein and mRNA signals showed a coherent detection in J-Lat 10.6^Activation+/Probe+^ after latency reversal whereas little or no signal was detected in J-Lat 10.6^Activation-/Probe+^ and J-Lat 10.6^Activation+/Probe-^. Using dilution series of activated J-Lat 10.6 cells in the parental Jurkat cell line, LOD was measured by automatic cell counting and analysis for dual recognition of HIV^mRNA+/GFP+^. LOD for RNAscope was significantly reduced to 45 NPM compared to RNAflow (**Figure 4B**). The NIS element software allowed for automated wide-field analysis and quantification of the proportion of cells with on-going viral replication, thereby determined as reactivation of latency (**Supplementary Figure 2**). Automated detection removed any bias incorporated due to human errors and thus contributed to a highly, unbiased detection system as all mRNA signal are related to cells that have an active transcription of GFP, and total number of cells based on detected nuclei following DAPI staining.

### RNAscope to Detect Diverse HIV-1 Subtypes

**FIGURE 5:**
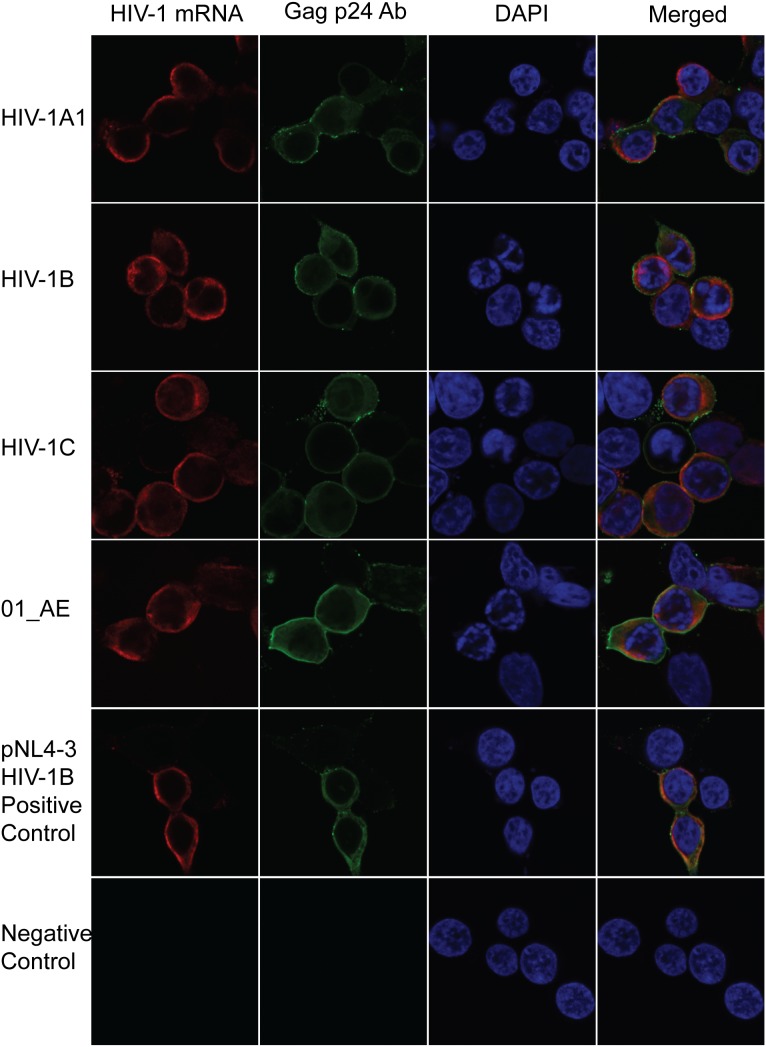
Subtype specificity of RNAscope using patients derived chimeric viruses of HIV-1 subtypes. Chimeric viruses having HIV-1A1, B, C and CRF 01_AE gag-pol recombinant plasmids were transfected in 293T cells. HIV-1 mRNA and p24 Gag protein was detected 24 h post-transfection by RNAscope and antibody staining.

Within the HIV-1 genome, the *gag-pol* region is known to be structurally, and evolutionary semiconserved (Li et al., 2015). HIV-1 has a high mutational rate, and thereby large variations occur on a genetic level between the subtypes. The original probe set was designed for HIV-1B subtype. To investigate if these probes could detect viral mRNA from various HIV-1 subtypes, HEK 293T cells were transfected with pNL4-3*gag-pol* clones of HIV-1A1, HIV-1B, HIV-1C, and 01_AE isolated from patients. The HIV^RNA+^ detection was complemented with immunofluorescence (IF) detection of the viral protein p24. This protein is translated early on during HIV-1 replication thereby making it a good validating marker for activation of latency. HIV^RNA+/p24+^ dual detection allowed for combinational validation of each cell harboring translation-competent virus. The probes were capable of detecting all subtypes using a standardized transfection for 24 h (**Figure 5**). Thus, these probes target a region that is semiconserved within the HIV-1 genome, indicating the capability of utilizing this method to quantify the proportion of latently infected cells in patient material independent on subtype.

### Detection of Translation-Competent Latently Infected Cells *ex vivo* Using RNAscope

**FIGURE 6:**
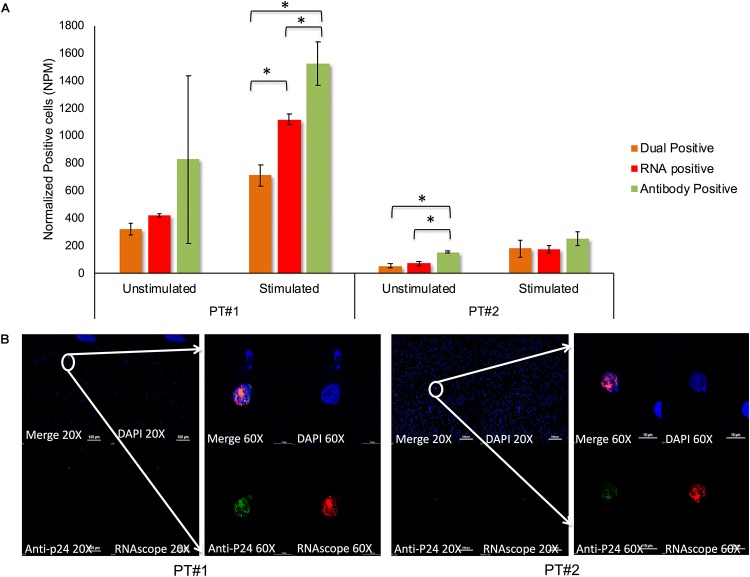
Counting of translation-competent cells in patients derived PBMC by RNAscope. **(A)** *Ex vivo* detection of HIV-1 mRNA and p24 protein in clinical samples using PBMCs from two therapy-naïve individuals with and without cLRA activation. After cLRAs stimulation, detectable HIV^mRNA+/p24+^ cells increased to 710 and 184 in Patient#1, Patient#2 indicating a reactivation of latent virus within the clinical samples. Patient 1 was infected with CRF01_AE viruses and had plasma viremia 1970000 copies/mL while patient 2 was infected with HIV-1C viruses with plasma viremia 69700 copies/mL. *p* < 0.05 are marked with ‘^∗^’ and consider significant. **(B)** Wide-field analysis, all HIV^mRNA+/p24+^ positive cells could be detected as single cells exhibiting viral mRNA correlating with anti-p24 detection indicating the applicability of the method at single-cell level.

*In vitro* models never wholly convey the inherent complexity of a biological system. Therefore, we aimed at evaluating HIV-1 reactivation from latency in clinical samples *ex vivo* using PBMCs from two therapy-naïve individuals. In the absence of latency reversal 319 and 54 cells/million PBMCs were HIV^mRNA+/p24+^ in Patient#1 and Patient#2, respectively, after normalizing the background with an HIV-negative control using RNAscope (**Figure 6A**). After LRAs stimulation, the number of detectable HIV^mRNA+/p24+^ cells increased to 710 and 184 in Patient#1 and Patient#2, respectively, indicating a reactivation of latent virus within the PBMC (**Figure 6A**). The HIV^mRNA+^ cells were found more frequently than dual (HIV^mRNA+/p24+^) positive cells but in a lower frequency than HIV^p24+^. In one patient-sample HIV^p24+^ cells were detected in a more substantial amount than HIV^mRNA+^ cells, but a higher standard deviation was found compared to HIV^mRNA+^ cells. As individual patients are known to have different proportions of latently infected cells, detectable HIV mRNA varied to a high degree between our two patients. A high percentage of viral mRNA could be caused by a large number of latently infected cells in therapy naïve individuals or by a high efficacy of the LRAs promoting ongoing viral replication. By wide-filed analysis, all HIV^mRNA+/p24+^ positive cells could be detected as single cells exhibiting viral mRNA correlating with anti-p24 detection (**Figure 6B**) indicating the applicability of the method at single-cell level. The capacity for detecting HIV^mRNA+/p24+^ cells in clinical samples indicates a translational potential of this method from *in vitro* to *ex vivo* applications.

## Discussion

In this study, the RNAflow and RNAscope techniques were evaluated using LRAs to induce the HIV-1 latent reservoir in a small volume of input cells. Our modified RNAscope performed better that RNAflow due to a limited cells loss and a lower detection limit of 45 cells/million cells. Although the probes for RNAscope were developed for HIV-1B, they enabled the detection of several other patient-derived HIV-1 subtypes (HIV-1A1, HIV-1B, HIV-1C, 01_AE). The method can also allow single cell characterization of HIV-1 RNA and protein simultaneously.

The fluorescence *in situ* hybridization-flow cytometry technique have been reported to be sensitive and specific in determining HIV-1 mRNA and protein after latency reactivation at the single-cell level. (Martrus et al., 2016; Baxter et al., 2017; Grau-Exposito et al., 2017). Earlier, the RNAflow studies have reported to use 1 x 10^6^ cells (Martrus et al., 2016). However the 18 washing steps needed contributed to a substantial loss of cells (∼70–82%), which is in line with previous studies which observed nearly a 70% cell loss (Baxter et al., 2017; Grau-Exposito et al., 2017). Therefore, final cells for analysis ended up to be around 2 × 10^5^ to 3 × 10^5^ from 1 × 10^6^ cells as starting material. This large proportion of cell loss is an important factor for the compromised lower detection limit for RNAflow which makes it in the present form of limited value to study the reservoir *ex vivo*. In contrast, we managed to get a higher sensitivity for the RNAScope. A recent study showed simultaneous detection of multiple viral nucleic acid intermediates and proteins using RNAscope at a single-cell resolution (Puray-Chavez et al., 2017). Here we evaluated the same protocol but with surface modification of the coverslips for non-adherent J-Lat cells and PBMCs which reduced the cell loss to less than 20%. Therefore we were able to scan nearly 8 × 10^5^ in the assay which contributed to a lower detection limit of 45 cells/million cells, 10-fold lower than RNAflow.

Among the several challenges of HIV-1 cure research, high-throughput drug screenings are essential to identify new promising LRAs efficiently. Also, the effect of combinations of LRAs or compounds that target multiple pathways controlling latency can be explored to identify the viral re-activation process (Rasmussen and Lewin, 2016). However, studies on *ex vivo* latency reversal, of LRAs targeting different cellular pathways, have given contradicting results. Often LRAs reactivate HIV-1 nonuniformly across different cells models (Spina et al., 2013) and frequently two drugs in combination increase the viral reactivation. E.g., a combination of PMA and Ionomycin increased the viral reactivation *ex vivo* when compared to PMA or Ionomycin alone (Spina et al., 2013; Baxter et al., 2017; Grau-Exposito et al., 2017). However, in our study we excluded PMA since it has been shown that PMA induction of HIV-1 replication can be Tat-independent (Luznik et al., 1995) and false results can be obtained when using PMC (Spina et al., 2013). An earlier study indicated that PKC agonists reactivated latent HIV-1 uniformly across different cell models (Spina et al., 2013). Our data showed that the PKC agonstists hTNF-α and prostatin gave slightly better reactivation in all three cell lines. The combination was also able to reactivate the viral reservoir in patients PBMCs.

Another challenge in HIV-1 molecular assays is the genetic variation of the subtypes. Both RNAflow and RNAscope probe sets were developed for subtype B. To the best of our knowledge, earlier studies have not commented on the subtype specificities of these assays (Martrus et al., 2016; Baxter et al., 2017; Grau-Exposito et al., 2017; Puray-Chavez et al., 2017). We tested the RNAscope HIV-1B probe sets for patient-derived HIV-1A1, HIV-1B, HIV-1C, and 01_AE subtypes and were able to detect the viral mRNA for all the subtypes.

Our study has limitations that merit comments. First, RNAscope can detect as low as 45 cells/million, but the standard deviation near the lower detection limit is also high in the patient samples tested. However, more sensitivity can be achieved by scanning a large number of cells. Since the number of remaining latently HIV-1 infected cells are low after long-term successful cART (Joos et al., 2008), it is important to further improve the lower LOD. Second, though the RNAscope probe can detect all of our subtypes, we did not test for all subtypes circulating globally. Finally, the assay can identify transcription and translation competent provirus but may not indeed represent the replication-competent latent HIV-1 reservoir as we do not measure infectious virus. However, this assay can be combined with TZM-bl cell-based assay as described (Sanyal et al., 2017).

In summary, our study showed that RNAscope could be used to quantify the number of directly observed individual cells expressing HIV-1 mRNA following cLRA. However, method needs improvement in lower detection limit to be applicable for the reservoir quantification on patients with suppressive therapy. Therefore, it can be a useful tool for characterization of translation-competent HIV-1 latently infected cells at single-cell resolution in the fields of HIV-1 pathogenesis and viral persistence. This method can also be adapted for single-cell transcriptomics and proteomics studies.

## Data Availability Statement

All data supporting the findings in this paper are included in the main text and Supplementary Information. All relevant data are available from the authors upon request.

## Author Contributions

UN and WZ designed the study and experimental plan. WZ performed RNAflow and RNAscope standardization. SSA performed the RNAscope analysis with patients’ derived materials. WZ and SSA wrote the method and result section. UN wrote the introduction and discussion part of the manuscript. AS provided intellectual input, the clinical material, revised the manuscript with critical input. All authors approved the final version of the manuscript.

## Conflict of Interest Statement

The authors declare that the research was conducted in the absence of any commercial or financial relationships that could be construed as a potential conflict of interest.
