# Supplementary material for: Characterization of Inducible Transcription and Translation-Competent HIV-1 Using the RNAscope ISH Technology at a Single-Cell Resolution
Source: Front Microbiol. 2018 Oct 2;9:2358. doi: 10.3389/fmicb.2018.02358 (PMC6176121; doi:10.3389/fmicb.2018.02358)
Supplement: Supplementary file 1 [file Data_Sheet_1.docx]

Supplementary Material

Characterization of inducible translation-competent HIV-1 using the RNAscope ISH technology at a single-cell resolution

**Wang Zhang,^1,2^ Sara Svensson Akusjärvi,^1^ Anders Sönnerborg,^1,3^ Ujjwal Neogi^1*^**

*** Correspondence:** Ujjwal Neogi, [ujjwal.neogi@ki.se](mailto:ujjwal.neogi@ki.se)


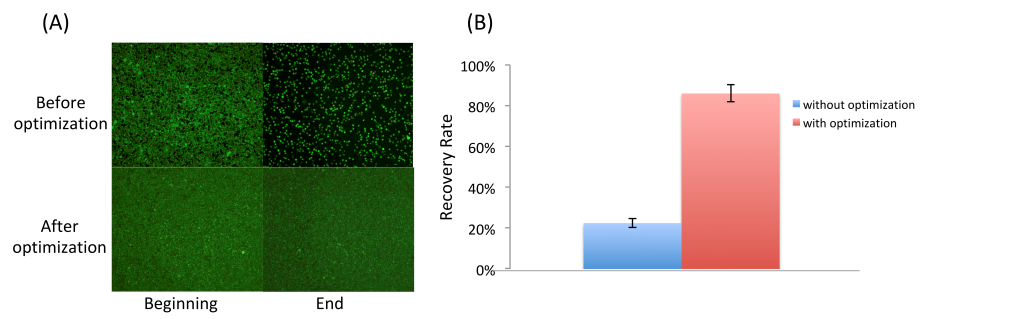


## Supplementary Figure 1. Optimized RNAscope workflow to improve cell recovery rate. (a) Poly-L-Lysine pre-treatment positively affected the cell coverage seen by an increase in the attachment of activated J-Lat 10.6 cells, measured by GFP. (b) The optimization induced an increase in cell coverage from 22.4% to 85.9%, from the input number of cells after RNAscope protocol

**
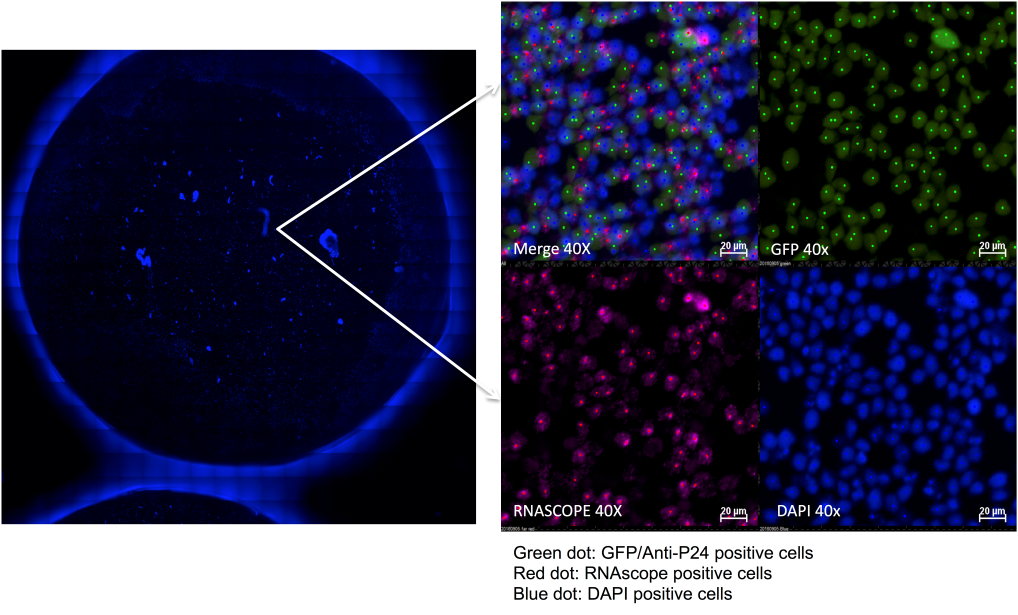
**

**Supplementary Figure 2. Automated counting of** HIV^mRNA+/GFP+,^ HIV^GFP+^ or HIV^mRNA+^ cells. The NIS element software allowed for automated wide-field analysis and quantification of the proportion of on-going viral replication, thereby determined as reactivation of latency. The single dot in the middle of cells was an identification mark added by the automatic function of NIS Element Software, that was identified as positive by the software.
